# Physiological and Environmental Regulation of Seed Germination: From Signaling Events to Molecular Responses

**DOI:** 10.3390/ijms23094839

**Published:** 2022-04-27

**Authors:** Emmanuel Baudouin, Juliette Puyaubert, Christophe Bailly

**Affiliations:** Laboratoire de Biologie du Développement, Institut de Biologie Paris-Seine (IBPS), Sorbonne Université, CNRS, UMR7622, F-75005 Paris, France; juliette.puyaubert@sorbonne-universite.fr (J.P.); christophe.bailly@sorbonne-universite.fr (C.B.)

A timely and efficient seed germination is critical for plantlets’ establishment and robustness as well as plant development and plant performance in both natural ecosystems and agrosystems. Therefore, the go/no-go decision for a seed to germinate is tightly controlled by a variety of endogenous and environmental signals perceived and integrated during seed imbibition or accumulated during the later stages of seed development. In particular, the initial switch from a non-germinating to a germinating state includes the release of dormancy, an inhibitory process established during seed maturation. Nevertheless, this switch can be reversed and dormancy reactivated (dormancy cycling) as long as environmental conditions are uncertain for proper seedling growth. In this scheme, environmental factors, e.g., temperature or water availability, appear central to triggering or restricting germination, and ongoing global warming will dramatically affect germination efficiency, and thereby species spreading, growth and final yield. Ahead of this major challenge for sustainable agriculture, the development of efficient strategies to mitigate the effects of climate change on the phenology of seeds is urgent and crucial.

To meet this challenge, it is essential to gain more understanding of the different levels of the regulation of seed germination and their spatiotemporal interplays, as well as their conservation in crops and non-model species. Indeed, recent major breakthroughs have significantly broadened our understanding of how germination is controlled. On the one hand, important advances on the molecular regulation of key actors have been achieved, as exemplified for DELAY OF GERMINATION-1 (DOG1), the most popular regulator of dormancy [1]. On the other hand, new directions have been explored, from which epigenetic regulation and endosperm–embryo crosstalk emerged as hot topics for future research [2,3]. Finally, the abundance of omics data paves the way to system biology-based approaches and the construction of integrative models.

This Special Issue gathers together eight articles (four research and four review articles) that question various facets of the regulation of seed development, seed dormancy and germination, which eventually impact seed capacity to germinate.

Hormonal metabolism and signaling are at the heart of the regulation of seed germination and, furthermore, control seed development, dormancy, vigor and longevity. Although these traits are regulated through complex interplays of many hormonal pathways, abscisic acid (ABA) and gibberellins (GA) remain the most important regulators and the balance between ABA and GA, the universal lever for the control of germination. In the present Issue, Sano and Marion-Poll [4] provide a comprehensive overview of ABA metabolism and its regulation during dormancy induction, dormancy release and germination. An emphasis is put on the transcriptional networks that regulate ABA metabolism throughout seed lifespan and the importance of epigenetic control that recently emerged. Through the case study of ABA metabolism, the authors also tackle important aspects of seed dormancy and germination control, e.g., the influence of maternal environment on seed progeny germination capacity or the interplay between environmental and hormonal factors. As presented in a final chapter, the contribution of natural inbred lines to uncover new regulators of ABA metabolism in seeds are examined.

In the recent years, important efforts have been dedicated to the identification of new genes/proteins involved in specific aspects of seed capacity to germinate and the unravelling of their function. In this light, Née et al. [5] investigated the function of *Arabidopsis thaliana Trx-y1* and *y2*, two y-type plastidial thioredoxins that exhibit antioxidant capacity. They report that *trx*-*y1* and *y2* mutations improve important seed traits, e.g., longevity or germination efficiency under water stress. Unexpectedly, no difference in seed reactive oxygen species (ROS) homeostasis is observed in mutant seeds and the authors propose that Trx-y participate in redox signaling rather than in ROS detoxification. Moreover, *trx-y* mutations impact the transcriptional regulation of abscisic acid (ABA) and gibberellin (GA) metabolisms. As redox-based signaling is central for seed dormancy release and germination [6], further characterization of the Trx-ys function in seeds is likely to shed light on new mechanisms linking ROS and hormone signaling.

The transitions from developing to dry mature and, eventually, to germinating seeds are accompanied by important changes in nuclear and chromatin organization, which reflect modifications in the global transcriptional activity, but also the necessary protection of genetic material during seed desiccation. The dynamics of DNA packaging are strongly dependent on the post-translational modifications (PTM) of histones and, possibly, the exchange between different histone variants. Consequences of histone PTM on seed dormancy and germination have been established but the dynamics and involvement of specific histone variants in this process are currently poorly documented. The study carried out by Layat et al. [7] provides valuable insights into the function of the histone chaperone HIRA which is required for H3.3 histone variant deposition. H3.3 is the predominant H3 variant in seed embryos and the lack of HIRA activity leads to histone H3 depletion and enhanced chromatin relaxation. Although viable, *hira* seeds exhibit a range of defects in relation to germination capacity. This study therefore establishes histone variant exchange as a new mechanism to regulate germination via the modification chromatin structure.

The specific functions of the different seed tissues, as well as their functional interactions, have been paid increasing attention and bridge seed development and seed germination processes over space and time [3]. These issues are addressed in a review article by Miray et al. [8] and in a research article by Sun et al. [9]. Miray and collaborators present an exhaustive overview of how oil metabolism is controlled in the endosperm during seed development [8]. In particular, they consider the regulatory mechanisms, especially at the transcriptional level, that underlie the differences in oil nature and content observed in endosperm and embryonic tissues. Importantly, they bring evidence that the functions of oils stored in endosperm exceed in fueling seedling metabolism and cover problematic aspects as diverse as seed dispersal, seed imbibition and the regulation of seed germination in relation to ROS signaling. For its part, the study by Sun and colleagues [9] revealed the specific gene expression profiles of height different tissues during early soybean seed development. The data obtained further evidence of the determinant weight of tissue specificity compared to developmental stage specificity in the determination of expression profile during seed development. They also give insights into the generic and specific gene expression trends between species and predict candidate transcriptional regulators operating in a tissue-specific or unspecific manner. This study therefore constitutes a step forward in our understanding of the contribution of individual tissues on seed development, which will pave the way towards integrated spatio-temporal models of transcriptome remodeling, from seed development to seed germination.

Recent studies have proven that mRNA metabolism is at the heart of the regulation of seed germination [2]. Indeed, the selective and dynamic modification, translation, storage and degradation of stored mRNAs are tightly controlled and determine whether the seed will remain dormant or will germinate. Nevertheless, the picture of the mRNA metabolism in germinating seeds is far from complete and how specific mRNAs are oriented to the one or the other process is currently unknown. Lou and colleagues [10] tackle these issues in a review dedicated to RNA-binding proteins (RBPs) and their emerging roles in seed biology. Hundreds of RBPs have been identified in plants and the review successively considers the functions of the four major RBP families, i.e., the PUF-type, DsRBD-type, GRPs and PPR-type, in relation to seed development and performance. As underlined by the authors, critical breakthroughs will now come from the identification of each RBP substrate mRNAs and the emergence of spatiotemporal models for RBP/mRNA interactions in seeds. They will also come from the elucidation of RBP regulation, in particular via PTMs, and its relationship with the signaling pathways controlling seed biology.

Although progresses in our understanding of the molecular mechanisms involved in germination control essentially come from the study of a handful of model plants and crops, the diversity of structure, germination behavior and responses to environmental signals of seeds among species requires broader studies, in particular towards wild and alternatively cultivated species. Indeed these plants represent unique opportunities to identify evolutionary-conserved regulatory networks and to investigate specific seed traits. In this Special Issue, a research paper by Pawłowski et al. [11] and a review paper by Cui et al. [12] provide new insights into the mechanisms controlling the germination of a wild species, wild rose, and an economically major species, oil palm, respectively. Wild rose seeds exhibit a combinational physiological/physical dormancy that is alleviated by warm and then cold temperature over several months and represents an obstacle for germination in field conditions. Using a proteomic approach, Pawłowski et al. [11] identified several proteins associated either with primary dormancy release or with secondary dormancy induction and discuss how these proteins may be related to dormancy regulation. As with wild roses, seed germination is a hurdle for oil palm cultivation. In their article, Cui et al. [12] provide the first comprehensive review of the specific aspects of oil palm seed germination and the metabolic pathways solicited. In particular, they underline the key role of haustorium which, as an interface with endosperm, controls reserve mobilization. They conclude with the importance of applying metabolomic and proteomic approaches to further investigations of oil palm seed metabolism and anticipate they will identify molecular markers to assist oil palm breeding.

The studies compiled in this Special Issue further illustrate that the control of germination integrates diverse levels of regulation, including chromatin organization, gene transcription, mRNA translation and protein post-translational modification resulting in a fine-tuned regulation of the seed metabolism. A major challenge is now to gather these fragmented pieces of information into a holistic and dynamic model integrating the different seed tissues and the regulation by endogenous and environmental cues.
